# The Expression Pattern of *p32* in Sheep Muscle and Its Role in Differentiation, Cell Proliferation, and Apoptosis of Myoblasts

**DOI:** 10.3390/ijms20205161

**Published:** 2019-10-18

**Authors:** Jianyu Ma, Caifang Ren, Hua Yang, Jie Zhao, Feng Wang, Yongjie Wan

**Affiliations:** Institute of Sheep and Goat Science; Nanjing Agricultural University, Nanjing 210095, China; 2017105027@njau.edu.cn (J.M.); 2014205007@njau.edu.cn (C.R.); 2018205011@njau.edu.cn (H.Y.); 2017105036@njau.edu.cn (J.Z.)

**Keywords:** *p32*, muscle development, myoblasts, differentiation, proliferation, apoptosis, *AMPK*

## Abstract

The complement 1q binding protein C (*C1QBP*), also known as *p32*, is highly expressed in rapidly growing tissues and plays a crucial role in cell proliferation and apoptosis. However, there are no data interpreting its mechanisms in muscle development. To investigate the role of *p32* in sheep muscle development, an 856 bp cDNA fragment of *p32* containing an 837 bp coding sequence that encodes 278 amino acids was analyzed. We then revealed that the expression of *p32* in the longissimus and quadricep muscles of fetal sheep was more significantly up-regulated than expression at other developmental stages. Furthermore, we found that the expression of *p32* was increased during myoblasts differentiation in vitro. Additionally, the knockdown of *p32* in sheep myoblasts effectively inhibited myoblast differentiation, proliferation, and promoted cell apoptosis in vitro. The interference of *p32* also changed the energy metabolism from Oxidative Phosphorylation (OXPHOS) to glycolysis and activated AMP-activated protein kinase (*AMPK*) phosphorylation in sheep myoblasts in vitro. Taken together, our data suggest that *p32* plays a vital role in the development of sheep muscle and provides a potential direction for future research on muscle development and some muscle diseases.

## 1. Introduction

Skeletal muscle accounts for about 40% of the body’s weight, and has many functions, such as maintaining energy requirements, maintaining posture, and protecting soft tissues. The normal development of skeletal muscle is a prerequisite for animals to maintain normal life activities and metabolism, and any abnormal development will lead to disease [1]. The fiber type of sheep muscle is closer to that of humans than that of mice [2]. Therefore, sheep may be a more suitable model animal for studying skeletal muscle [2]. The growth and development of sheep skeletal muscle are also closely related to meat production. The development of skeletal muscle is complicated, including the formation and proliferation of myoblasts, the formation of myotubes and muscle fibers, and the final maturation process [3,4]. The proliferation of myocytes and the formation of muscle fibers are mostly completed in the fetal period [5]. Thus, the fetal period is a critical period of skeletal muscle development. The proliferation of sheep myofibers occurs before or around 100 days of gestation, and then myofibers grow to fuse together and experience hypertrophy [6]. In our previous study, the RNA-seq data showed that the expression of *p32* in the longissimus muscle of fetal sheep was significantly higher than that in postnatal sheep muscle [7], so we hypothesized that *p32* may play a crucial role during the skeletal muscle development of sheep.

The complement 1q binding protein C (*C1QBP*) (also known as *p32*), the hyaluronic acid binding protein 1 (*HABP1*), and the receptor for the globular head domains of complement C1q (*gC1qR*) [8], are conserved proteins primarily localized in the mitochondrial matrix [9] but also expressed in other subcellular compartments, including the nucleus, endoplasmic reticulum, Golgi, and cell surface [10]. Some studies have suggested that *p32* is highly expressed in metabolically active and rapidly growing tissues, such as tumors of the breast, epidermis, and ovary [11,12,13,14,15]. The *p32* protein plays an important role in maintaining oxidative phosphorylation (OXPHOS) [16]. The knockdown of *p32* in human cancer cells strongly affects OXPHOS enzyme levels and activity and shifts their metabolism from OXPHOS to glycolysis. Moreover, *p32* plays an important role in cell proliferation, adhesion, migration, and invasion [13,17,18]. The expression of *p32* in the placenta during pre-pregnancy was significantly higher than that in the late pregnancy, and its expression in the trophoblast was significantly reduced in the case of fetal growth restriction in women [8]. The *p32*-deficient mice exhibited severe embryonic developmental defects, and the knockdown of *p32* in mouse embryo fibroblast (MEF) cells significantly reduced ATP production and delayed cell proliferation [19]. Infants with biallelic *C1QBP* mutations presented with cardiomyopathy accompanied by multisystemic involvement (liver, kidney, and brain), and children and adults presented with myopathy. They all present with multiple OXPHOS deficiencies [20].

Notably, the AMP-activated protein kinase (*AMPK*) was more significantly phosphorylated in the hearts of *p32*-deficient mice compared to the controls [21]. *AMPK* is a highly conserved sensor of cellular energy status that could be activated under low intracellular ATP conditions [22] and is involved in cell growth, proliferation, apoptosis, autophagy, and other basic biological processes [23]. Liver Kinase B1 (*LKB1*) is the upstream activating kinase of the stress-responsive *AMPK* and acts as a low-energy checkpoint in cells [24]. In addition, *AMPK* responds to energy stress by suppressing cell growth, in part through its inhibition of the rapamycin-sensitive mTOR (*mTORC1*) pathway [25]. Indeed, *mTORC1* is an important regulator during embryonic and adult myogenesis, and an *mTORC1* deficiency in muscle stem cells affects injury-induced muscle regeneration [26].

*p32* is highly expressed in rapidly growing tissues, such as the skeletal muscle of fetal sheep. However, the effects of *p32* on sheep muscle development and whether it activates the *AMPK* signaling pathway remain unknown. Therefore, our study aimed to investigate the role of *p32* on the muscular development of sheep. We cloned the *p32* coding sequence of sheep and examined the expression of *p32* in the longissimus muscle and quadriceps muscle of Hu Sheep at different developmental stages. The effect of *p32* on the proliferation, differentiation, and apoptosis of sheep myoblasts was investigated by transfecting siRNA into Hu sheep myoblasts isolated in vitro to interfere with the expression of *p32* in myoblasts. In addition, changes in *AMPK*-associated genes were investigated to further reveal the link between them. This study lays the foundation for exploring the role of *p32* in muscle development and its potential mechanisms.

## 2. Results

### 2.1. cDNA Cloning and Sequence Analysis of p32

**Figure 1:**
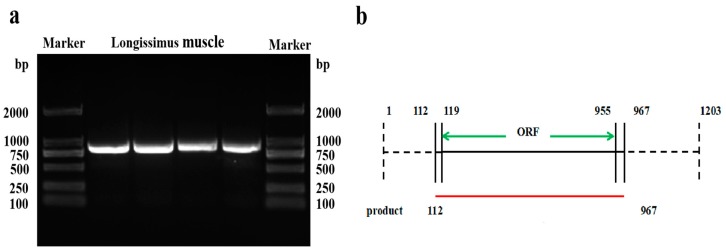
The *p32*-coding sequence *(p32*-CDS) primers were designed for amplifying the open reading frame (ORF) of *p32* and the sketch of the *p32*-CDS amplification product from Hu sheep (**b**). The four lanes represent *p32*-CDS amplification product using the Longissimus muscle’s cDNA (**a**). The dashed line represents the untranslated region of *p32*, and the green line represents the ORF of *p32*.

**Figure 2:**
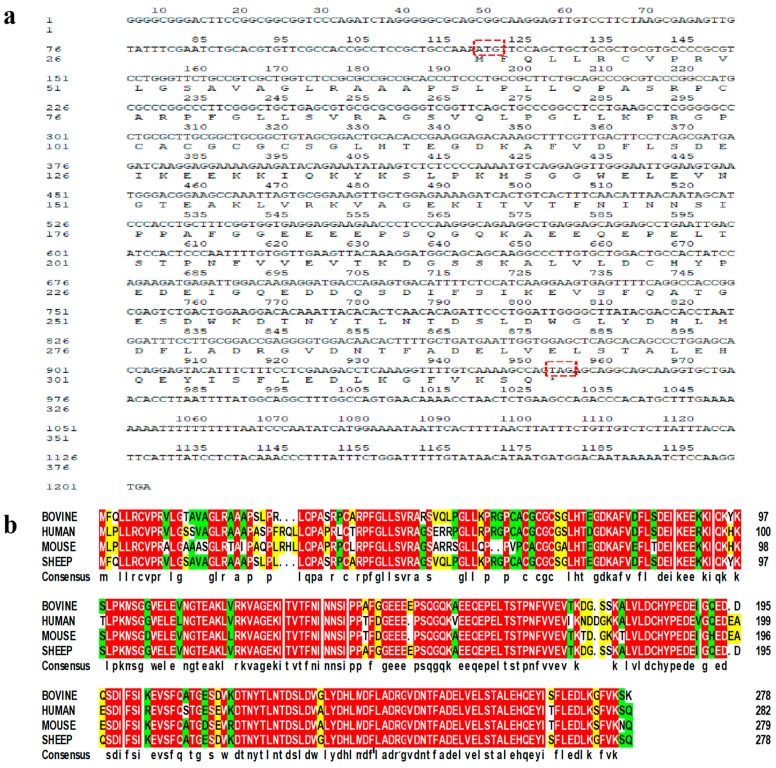
The cDNA fragment and predicted protein sequences of ovine *p32*-CDS are also shown. The start and stop codons are framed in red (**a**). The protein sequence was predicted by the BLAST tool of DNAMAN (version 6.0, LynnonBiosoft, San Ramon, CA, USA) and is presented under the coding sequence. * means the translation is terminated. (**a**) Comparison of the bovine, mouse, and human *p32* amino acid sequences. The *p32* amino acid sequence was aligned to those of bovines, murines, and humans by DNAMAN (**b**). The same amino acid residues in four, three, and two species are highlighted in red, green, and yellow, respectively, while the amino acid residues present in only one species are highlighted in white. The apostrophes indicate that these amino acid sequences are absent.

The cDNA fragment of the *p32* CDS was successfully obtained by PCR amplification (Figure 1a). Sequence analysis showed that the coding sequence of *p32* was 837 bp, encoding a 278-amino acid protein with a predicted molecular weight (MW) of 32 kDa (Figure 1b). Sequence alignments indicated that the amino acid sequence of the Hu sheep *p32* that we obtained is highly homologous to other species. It has 96.82% homology with Bos taurus amino acids (NCBI reference, number NM_001034527), 84.81% similarity with human amino acids (NCBI reference number XP_006520664), and shares 81.63% of its identity with mouse amino acids (NCBI reference number NM_007573) (Figure 2b).

### 2.2. The Expression Level of p32 in Hu Sheep Longissimus Muscle Tissues and Quadricep Muscle Tissues

**Figure 3:**
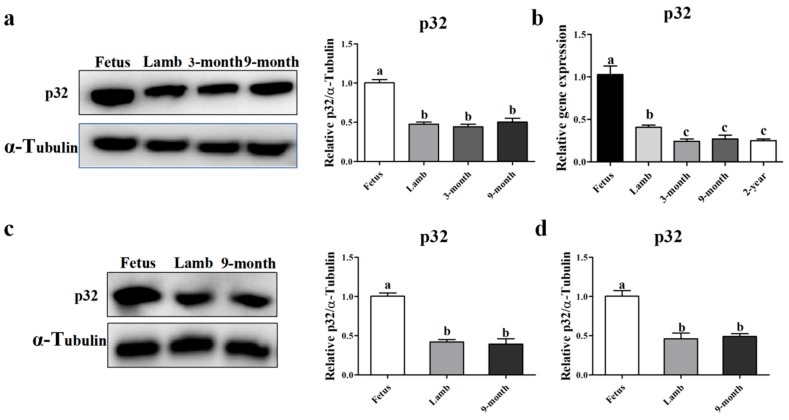
Expression patterns of *p32* protein (**a**) in the longissimus muscle of Hu sheep at different developmental stages (fetus, lamb, 3 months old, and 9 months old) were measured by Western blot. Expression of *p32* mRNA (**b**) in the longissimus muscle of Hu sheep at different developmental stages (fetus, lamb, 3 months, 9 months, and 2 years) were measured by qRT-PCR. Expression patterns of the *p32* protein (**c**) and mRNA (**d**) in the quadriceps muscle of sheep at three different developmental stages (fetus, lamb, and 9-month) were measured by qRT-PCR and Western blot, respectively. Expressions of the gene were normalized to glyceraldehyde-3-phosphate dehydrogenase (*GAPDH*) and related to the fetus. Results are expressed relative to the fetus as mean values ± SEM (*n* = 3). a, b, c: different letters denote statistically significant differences within each group; *p* < 0.05.

The expression of *p32* mRNA and protein in the longissimus muscle and quadriceps muscle at different developmental stages was detected by Western blot analysis (Figure 3a,c) and qRT-PCR (Figure 3b,d). The expression level of *p32* in fetal sheep’s longissimus muscle and quadriceps muscle was significantly higher than in other developmental stages (*p* < 0.05). This result suggests that *p32* plays an important role in fetal muscle.

### 2.3. The Expression Level of p32 During Myoblast Differentiation in Vitro

**Figure 4:**
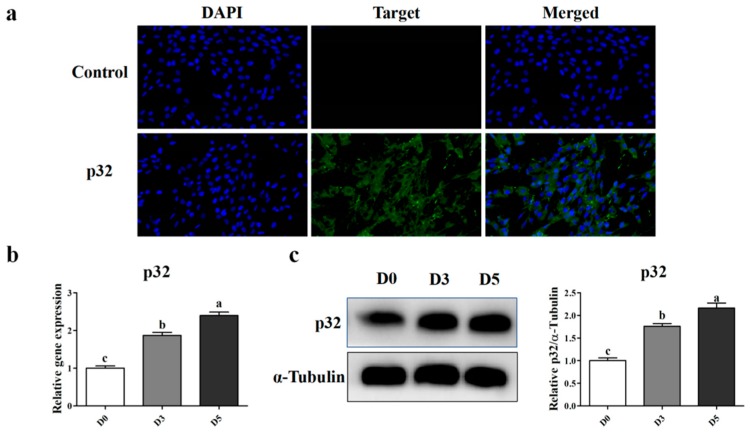
Immunofluorescence analysis of the *p32* protein in sheep myoblasts (**a**). Sheep myoblast stain with the *p32* antibody (green) and nuclear stain DAPI (blue). Scale bar = 100 μm. The expression of the *p32* mRNA (**b**) and protein (**c**) during myoblasts (and differentiated into myotubes) was detected by qRT-PCR and Western blot. The expression was normalized to glyceraldehyde-3-phosphate dehydrogenase (*GAPDH*) and related to the D0. Results are expressed relative to the D0 as mean values ± SEM (*n* = 3). a, b, c: different letters denote statistically significant differences within each group; *p* < 0.05.

To investigate the role of *p32* in muscle development, we isolated sheep myoblasts [6], and the Immunofluorescence analysis showed that *p32* was located in the cytoplasm of myoblasts (Figure 4a). In order to better localize *p32*, we isolated the mitochondria of myoblasts and compared the expression levels of *p32* in mitochondria and cytoplasmic protein without mitochondria. As shown in Figure S1, the expression of *p32* in mitochondria was significantly higher than in other cellular tissues. Furthermore, the expression of *p32* during myoblast differentiation was detected using qRT-PCR (Figure 4b) and Western blot (Figure 4c). The results showed that the expression of *p32* in myotubes was significantly higher than that in myoblasts.

### 2.4. The Interference of p32 Inhibits Myoblast Differentiation in Vitro

**Figure 5:**
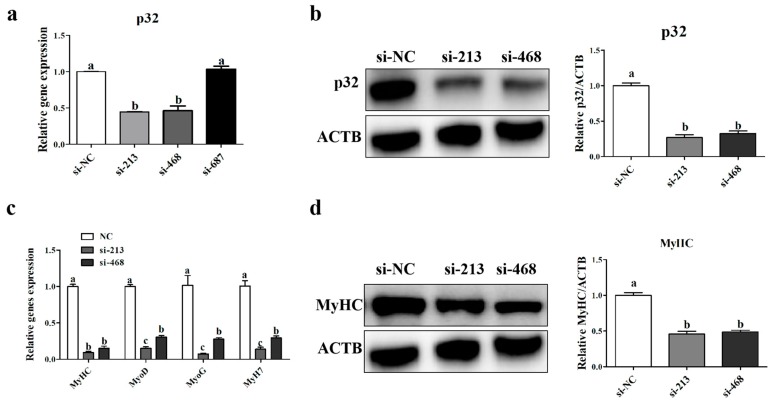
The expression of the *p32* mRNA (**a**) of myoblasts was determined at 24 h post-transfection, with the siRNAs targeting *p32* (si-213, si-468, si-689, and non-targeting control siRNA (si-NC)) by qRT-PCR (**a**). The expression of the *p32* protein was determined at 48 h after transfection with si-213, si-468 and si-NC using Western blot (**b**). The expression level of myosin heavy chain (*MyHC*), *MyoD*, myogenin (*MyoG*), and myosin heavy chain 7 (*MyH7*) mRNA was detected at 24 h after transfection with si-213, si-468, and si-NC by qRT-PCR (**c**). The expression of the *MyHC* protein was determined at 48 h after interference p32 using Western blot (**d**). Expression of gene and protein were normalized to β-actin (*ACTB*) and related to si-NC as mean values ± SEM (*n* = 3). a, b, c: different letters denote statistically significant differences within each group; *p* < 0.05.

To validate whether *p32* plays a role in myoblast differentiation, we transfected sheep myoblasts with *p32* siRNA. The results indicated that the interference efficiency of si-213 and si-468 were higher than that of si-687 (*p* < 0.05, Figure 5a). The Western blot also showed that the si-213 can significantly reduce the expression of the *p32* protein (*p* < 0.05, Figure 5b). The expression of the myogenic differentiation 1 (*MyoD1)*, myogenin (*MyoG*), myosin heavy chain (*MyHC*), and myosin heavy chain 7 (*MyH7*) mRNA, which are myoblast differentiation marker genes, were detected by qRT-PCR (Figure 5c). The expression of the *MyHC* protein was detected by Western blot (Figure 5d). The results indicated that the interference of p32 reduced the expression of *MyoD*, *MyoG*, *MyHC*, and MyH7 (*p* < 0.05, Figure 5c,d). To further verify this result, we induced myoblast differentiation at 48 h after transfection, and the total fusion index was detected at 5 days after differentiation using immunofluorescence of *MyHC*. As shown in Figure S2, interference of *p32* significantly decreased total fusion index of sheep myoblasts (*p* < 0.05). This result suggests that the interference of *p32* inhibits myoblast differentiation in vitro.

### 2.5. The Interference of p32 Inhibits the Cell Proliferation of Sheep Myoblasts Cultured in Vitro

**Figure 6:**
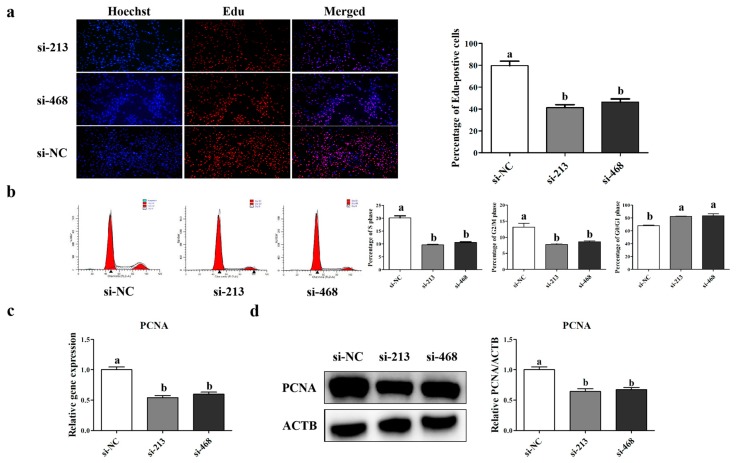
Cell proliferation was determined using EDU after transfection with si-213, si-468, and si-NC (**a**). Nuclear stain Hoechst (blue) and the proliferating cell stain EDU (red). The effect of siRNA transfection on the cell cycle progression in myoblasts (**b**). DNA content levels are also represented (b). The expression level of proliferating cell nuclear antigen (*PCNA*) mRNA (**c**) and protein (**d**) was measured by qRT-PCR and Western blot. The expression of genes and proteins was normalized to β-actin (*ACTB*) and related to si-NC. a, b, c: different letters denote statistically significant differences within each group; *p* < 0.05.

In order to examine the effects of *p32* on myoblast proliferation, we examined the proliferation of myoblasts after interference with si-NC, si-213, and si-468. At 36 h after transfection, we found that knocking down *p32* could inhibit cell proliferation (*p* < 0.05, Figure 6a) in myoblasts and shift the cell cycle (Figure 6b). After transfection with siRNA-213 and si-468 respectively, a significant decrease in the percentage of cells in the S and G2/M phases was observed. (*p* < 0.05, Figure 6b). The percentage of G0/G1 phase cells increased in the *p32*-deficient myoblasts (*p* < 0.05, Figure 6b). To confirm these results, the expression level of the cell proliferation related gene, proliferating cell nuclear antigen (*PCNA*), was detected by qRT-PCR and Western blot. The results showed that the expression levels of *PCNA* mRNA and the protein were decreased after transfection with siRNA-213 and si-468 respectively (*p* < 0.05, Figure 6c).

### 2.6. The Interference of p32 enhances the Cell Apoptosis of Sheep Myoblasts Cultured in Vitro

**Figure 7:**
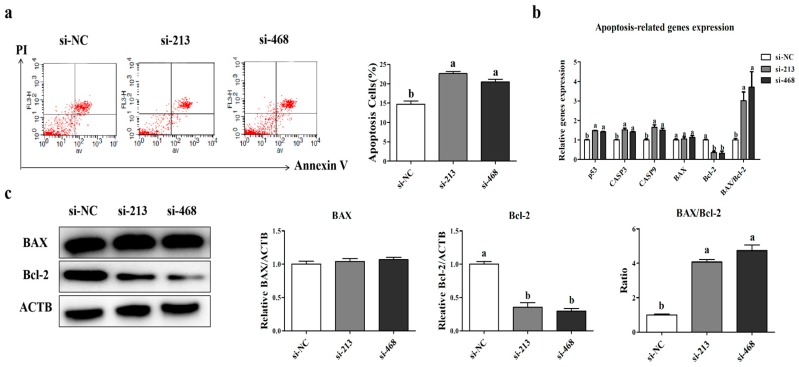
Apoptosis rates after transfection were determined by flow cytometry (**a**). Apoptosis-related gene expression (**b**) and apoptosis-related protein expression (**c**) in the transfected myoblasts were determined using qRT-PCR and Western blot, respectively. The expression of genes and proteins was normalized to β-actin (*ACTB*) and related to si-NC as mean values ± SEM (*n* = 3). a, b, c: different letters denote statistically significant differences within each group; *p* < 0.05.

Flow cytometry analysis was performed to detect the apoptosis rates in the control and siRNA interference groups, and the results showed a significant increase in the apoptosis rate of myoblasts after the transfection of *p32* siRNA (*p* < 0.05, Figure 7a). To further confirm this result, the expression levels of the apoptosis-related genes include *p53*, *CASP3*, *CASP9*, Bcl-2-associated X protein (*BAX*), and B-cell lymphoma 2 (*Bcl-2*). The ratio of *BAX/Bcl-2* was quantitated by qRT-PCR. The results indicated that the expression of these pro-apoptotic genes (excluding *BAX*) was increased, while the anti-apoptotic gene *Bcl-2* was decreased (*p* < 0.05, Figure 7b). Meanwhile, the trend of the *BAX* and *Bcl-2* protein expression levels was consistent with their mRNA (Figure 7c). These results showed that *p32*-siRNA could effectively promote myoblast apoptosis.

### 2.7. The Interference of p32 Shifts Energy Metabolism from OXPHOS towards Glycolysis in Sheep Myoblasts in Vitro

**Figure 8:**
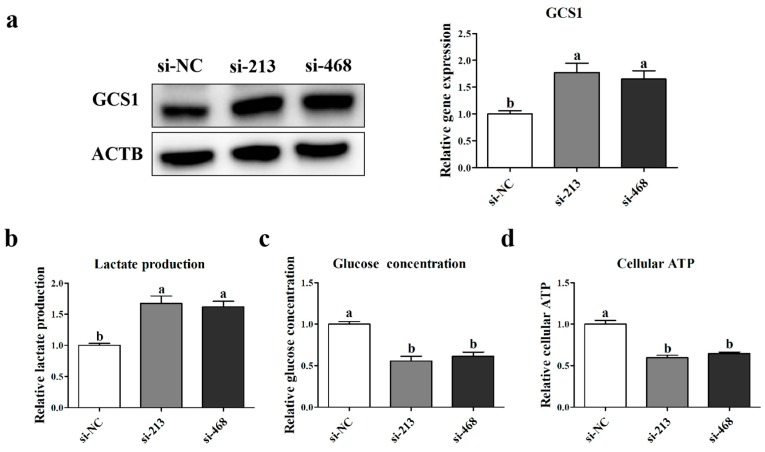
The expression of *GCS1* protein was detected using Western blot (**a**). Lactate and glucose concentrations in the cell media (**b**,**c**) and cellular ATP (**d**) were determined using an ELISA assay (**a**). Expression of the protein was normalized to β-actin (*ACTB*) and related to si-NC. Results are expressed relative to the si-NC as mean values ± SEM (*n* = 3). a, b, c: different letters denote statistically significant differences within each group; *p* < 0.05.

In order to verify the effects of *p32* on the energy metabolism of myoblasts, we detected the expression levels of the endoplasmic reticulum anchored enzyme mannosyl-oligosaccharide glucosidase I (*GCS1*) protein (Figure 8a), which regulates glucose metabolism with *p32*. The lactate and glucose concentration in the culture medium and the cellular ATP level after transfection were detected using an ELISA assay (Figure 8b–d). The results showed that the p32 knockdown in myoblasts significantly increased lactate production and glucose consumption (*p* < 0.05, Figure 8) and simultaneously reduced the cellular ATP level (*p* < 0.05, Figure 8a). These results confirm our hypothesis that a lack of *p32* could shift the energy metabolism from OXPHOS to glycolysis.

### 2.8. Interference with p32 Activates AMPK Phosphorylation in Sheep Myoblasts

**Figure 9:**
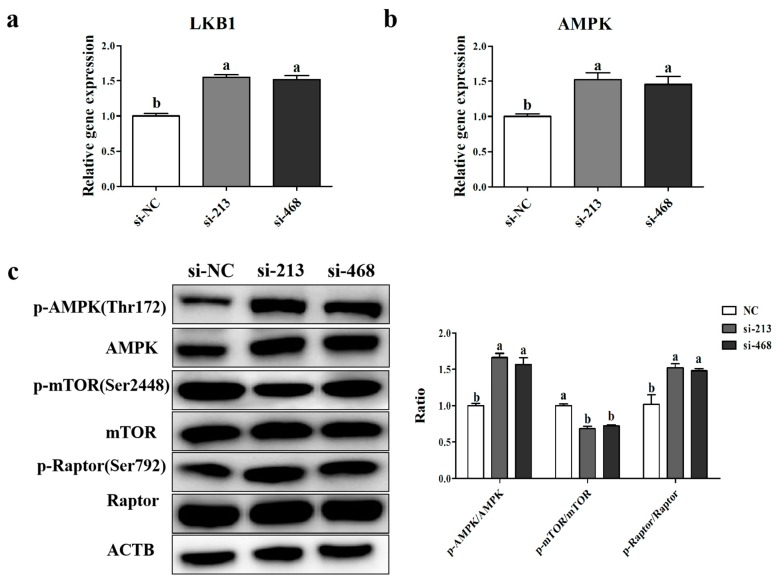
Liver Kinase B1 (*LKB1*) and AMP-activated protein kinase (*AMPK*) mRNA expression in the transfected myoblasts (**a**,**b**) was determined using qRT-PCR. *AMPK*, *p-AMPK*, *mTOR*, *p-mTOR*, *Raptor*, and *p-Raptor* protein expression (**c**) was determined using Western blot, and the ratios of these proteins are presented in column (**c**). The expression of genes and proteins was normalized to β-actin (*ACTB*) and related to si-NC as mean values ± SEM (*n* = 3). a, b, c: different letters denote statistically significant differences within each group; *p* < 0.05.

To verify whether the loss of *p32* could activate the *AMPK* signaling pathway, we detected, by qRT-PCR, the expression levels of *LKB1* and *AMPK* mRNA 24 h after transfection, and the ratios of *p-AMPK* (Thr172)/*AMPK*, *p-mTOR* (Ser2448)/*mTOR*, and *p-Raptor* (Ser792)/*Raptor* were also detected 48 h after transfection by Western blot. As shown in Figure 9b,c, after knocking down *p32*, the expression of the *LKB1* and *AMPK* genes in the myoblasts was significantly increased, and the ratios of *p-AMPK* (Thr172)/*AMPK* and *p-Raptor* (Ser792)/the regulatory associated protein of *mTOR* (Raptor) were also increased. Moreover, the ratio of *p-mTOR* (Ser2448)/*mTOR* was obviously decreased (Figure 9c). All these results suggest that the lack of *p32* can increase the expression of *LKB1*, activate *AMPK*, and then inhibit *mTORC1*. The activated *AMPK* inhibited the activity of *mTORC1*, ultimately leading to an increase in the apoptosis rate and an inhibition of cell proliferation.

## 3. Discussion

As the largest organ in the body, skeletal muscle not only provides protection for the mammalian motor system, but also provides a place for the glucose oxidation of surrounding tissues [6]. The normal development of skeletal muscle is a prerequisite for animals to maintain normal life activities and metabolism. Any abnormal development will lead to diseases, such as muscular dysplasia, muscle atrophy, and muscle hypertrophy [1]. The growth and development of skeletal muscle is a complex physiological process, which can be divided into four stages, including the formation and proliferation of myoblasts, the formation of myotubes and muscle fibers, and the final maturation process [3,4]. The development of skeletal muscle is inseparable from the precise regulation of many factors, many of which interact with genes involved in cell proliferation, differentiation, regeneration, migration, and apoptosis to form a complex and precise regulatory network to maintain the normal development of skeletal muscle [21,27].

Accumulating evidence has demonstrated the important role of *p32* in metabolically active and rapidly growing tissues, such as in the placenta and various tumors [8,11,12,13], and it is also highly expressed in the skeletal muscle of fetal sheep [7]. The fetal period is a key period of skeletal muscle development, as the proliferation of myocytes and the formation of muscle fibers are mostly completed in the fetal period [5,6,27]. Skeletal muscle fiber hyperplasia is completed during gestation and fixed at birth [28]. However, little research has been done on the function of *p32* in sheep muscle development. In this study, we cloned the sheep *p32*-CDS, and obtained the coding sequence of this gene. Then, we examined the expression of *p32* in the skeletal muscle of Hu Sheep at various growth periods in vivo and the expression of *p32* during myoblast differentiation in vitro. In addition, we found that the knockdown of *p32* in sheep myoblasts can inhibit differentiation and proliferation, thereby causing apoptosis. Moreover, we found that the knockdown of *p32* in myoblasts can reduce the cellular ATP level and activate the *AMPK* signaling pathway.

In the present study, we cloned the *p32*-CDS from the longissimus muscle of Hu Sheep. The results showed that the coding sequence of *p32* was 837 bp, encoding 278 amino acids. The *p32* coding sequence encodes 278, 279, and 282 amino acids in cattle, mice, and humans, respectively [29,30]. The amino acid sequence of sheep *p32* has high homology to the *p32* in cattle (96.82%) and is also homologous to mice (81.63%) and humans (84.81%). This result proves that the *p32* protein is highly conserved, and its function may be similar among various species, such as in human tumors and mice placentas [8].

The results of *p32* mRNA and protein expression in the longissimus muscle confirmed the results of previous studies [7]. Moreover, the expression of *p32* in the quadriceps of fetal sheep is also higher than in the quadriceps during other developmental stages. One of the characteristics of the skeletal muscle of fetal sheep is rapid growth [31], which is mainly based on the rapid proliferation and differentiation of myoblasts [5]. In addition, *p32* is necessary for fast-growing tissues [11,12,13,14,15]. Hence, we speculated that *p32* plays a key role in skeletal muscle development in fetal sheep. However, the mechanism of this phenomenon is still unclear.

To understand the role of *p32* in the development of muscle, we isolated sheep myoblasts. The results showed that *p32* was mainly expressed in the cytoplasm by immunofluorescence, which was consistent with the results in MEFs [19], MDA-MB-231 cells, and other cancer cells [14,32]. To further analyze the localization of *p32* in myoblasts, we measured the expression of *p32* protein in mitochondria and it in other cellular organizations. The results indicate that *p32* is mainly present in mitochondria, but it is also expressed in other organelles. The localization of *p32* in myoblasts is similar in cancer cells [9,10] Then, we differentiated myoblasts into myotubes in vitro, and the expression of *p32* was measured during differentiation. The results showed that the expression level of *p32* was increased according to the days of differentiation. These results indicated that *p32* could promote myoblast differentiation. To verify this hypothesis, three *p32*-specific siRNAs were used to inhibit the expression of *p32* in myoblasts, and we selected si-213, which has the best interference effect, for subsequent experiments. *MyHC* and *MyoD*, and its family genes, *MyoG* and *MyH7*, are marker genes for myoblast differentiation, as they regulate and initiate the fusion and differentiation of myoblasts [33]. In the present study, the expression of the *MyHC*, *MyoD*, *MyoG*, and *MyH7* mRNA and the *MyHC* protein was decreased after transfection. These results suggested that *p32* plays a crucial role during myoblast differentiation in vitro. However, the expression pattern of *p32* in longissimus muscle was opposed to its expression during myoblasts differentiation. Development of fetal muscle is very complicated, and many factors are involved in regulating this process. The difference in *p32* expression pattern between in vivo and in vitro remains unclear and it will be studied in further study.

Myoblast proliferation and apoptosis are also important for muscular development [34]. Some studies have shown that the loss of *p32* in tumor cells and cytotrophoblasts can affect cell proliferation and apoptosis [8,32]. Thus, we speculated that a low or deficient expression of *p32* leads to cell apoptosis and slows the cell proliferation of sheep myoblasts. The flow cytometry results showed that the knockdown of *p32* in myoblasts could significantly increase the apoptosis rate and change the cell cycle. Cell cycle control represents a major regulatory mechanism for cell proliferation. Our results suggested that the interference of *p32* can increase G0/G1-phase cells and induce S-phase arrest. In the present study, the knockdown of *p32* in myoblasts increases the mRNA and protein expressions of apoptosis-related genes, such as *Caspase3*, *p53* and the ratio of *BAX*/*Bcl-2*. In addition, the lack of *p32* in myoblasts also decreased the expression level of the proliferation marker gene *PCNA*. The EDU results also showed that *p32*-deficient myoblast proliferation was slower than that of the control group. All of these results indicate that the loss of *p32* in myoblasts could promote cell apoptosis and impair cell proliferation. Thus, *p32* is important for maintaining myoblast proliferation and apoptosis. However, the mechanism by which *p32* affects myoblast proliferation and apoptosis is unclear.

Skeletal muscle glucose metabolism is essential for maintaining glucose homeostasis [35]. When the balance of glycolysis and OXPHOS in skeletal muscle is broken, some diseases may occur. For example, the OXPHOS level in the skeletal muscle of patients with type 2 diabetes is much smaller than that of normal people, and the glycolysis is greater than that of normal people [35]. Thus, *p32* is necessary for maintaining normal levels of OXPHOS. The loss of *p32* affects OXPHOS enzyme levels and activities and shifts energy metabolism to glycolysis in tumor cell lines [33]. This is due in part to *GSC1* [36]. Glycolysis is a series of metabolic processes by which one molecule of glucose is catabolized to two molecules of pyruvate with a net gain of 2 ATP [37]. Pyruvate is then converted to lactic acid in animals. However, during OXPHOX, pyruvate can be further oxidized to CO_2_ and H2O in the mitochondria through the tricarboxylic acid (TCA) cycle and the respiratory chain. One molecule of glucose is metabolized to produce 32 ATP [37]. Thus, glycolysis is much less than the ATP produced by oxidative phosphorylation. In the present study, the expression of *GSC1* was increased after the knockdown of *p32*, and higher levels of glucose consumption and lactate production were observed in *p32*-deficient myoblasts. In addition, the knocking down of *p32* in myoblasts also decreased the cellular ATP level. These results indicated that a lack of *p32* in myoblasts could change the cellular metabolism from OXPHOS to glycolysis and reduce ATP production significantly.

The *AMPK* signaling pathway is activated under low cellular ATP level conditions [22] and is also involved in cell growth, proliferation, and apoptosis [23]. We hypothesized that knocking down *p32* led to a decrease in cellular ATP levels, which further activated the *AMPK* pathway, inhibited cell proliferation and differentiation, and promoted apoptosis. The *AMPK* signaling pathway is complicated [38]. *LKB1* is an upstream activation kinase of the stress-responsive AMP-activated kinase and acts as a low-energy checkpoint in cells [24,39,40]. *AMPK* directly inhibits *mTORC1* by phosphorylating the *mTORC1* binding partner, *Raptor* [25]. By inhibiting *mTORC1*, *AMPK* blocks the two major biosynthetic pathways required for cell growth: protein and RNA synthesis. The goal of the relationship between *AMPK* and *mTORC1* is to adjust the energy supply requirements of the anabolic process [41]. *mTORC1* is a central regulator of cell growth [42]. The loss of *mTORC1* slows but does not abolish myoblast proliferation and differentiation [26]. In the present study, the expression levels of *LKB1* mRNA and protein were higher in *p32*-deficient myoblasts. At the same time, *AMPK* and phosphor-*AMPK*(Thr172) mRNA expressions were also higher in *p32*-deficient myoblasts. These results suggested that the knockdown of *p32* in sheep myoblasts could activate the *AMPK* signaling pathway by increasing the expression of *LKB1*. Furthermore, this may be the result of changing the cellular metabolic pathways and reducing cellular ATP. In our study, the expression of *p-mTOR* (ser2448) was significantly decreased by the knockdown of *p32* in sheep myoblasts. Meanwhile, the expression of *p-Raptor* was increased in the interfered cells. All of the above results indicate that the knockdown of *p32* activates *AMPK* in myoblasts, thereby inhibiting the activity of *mTORC1*, and eventually inhibiting cell proliferation and enhancing cell apoptosis.

Although many studies have identified the functions of *p32*, this study primarily explored the role of *p32* in muscle development. However, we only studied the knockdown of *p32* on myoblasts in vitro. Subsequent studies can be performed in vivo and examine the overexpression *p32,* to investigate the effects on muscle development. Our results illustrate the importance of *p32* in muscle development and muscle glucose metabolism. *p32* may be a check point for some muscular developmental diseases and muscular metabolic diseases, such as muscular dysplasia and type 2 diabetes. However, this hypothesis needs to be further verified in subsequent studies.

## 4. Materials and methods

### 4.1. Sample Collection

All experimental procedures involving animals were approved and carried out in accordance with the relevant guidelines set by the Ethics Committee of Nanjing Agricultural University, China (Approval ID: SYXK2011-0036; date: 6 December 2011).

All sheep in this experiment were fed under the same conditions with natural light and free access to food and water at the Taizhou Hailun Sheep Industry Co., Ltd. (Taizhou, China). Longissimus muscle samples were taken between the 12th and 13th thoracic vertebrae to ensure the same part of each sheep was obtained from the nine Hu rams at the fetus, lamb, and adult stages (*n* = 3 at each stage). All samples were washed in physiological saline five times to minimize blood contamination. The tissue samples were fixed with a Bouin fixative for 24 h and embedded in paraffin for immunohistochemistry. They were then collected with RNAlater and snapped frozen in liquid nitrogen immediately for RNA and protein extraction.

### 4.2. The Isolation, Purification, and Culture of Sheep Myoblasts.

According to previous studies, sheep myoblasts were isolated by a two-step enzymatic method using muscle from newborn 5-day-old lambs [7]. Briefly, leg muscles were cut into small pieces and washed three times with DPBS, digested with 0.1% type I collagenase (Sigma-Aldrich, Saint Louis, MO, USA) for 1 h, and then digested with 0.25% trypsin (Gibco, Grand Island, NY, USA) for 30 min. The tubes were shaken every 10 min and filtered through a 200-mesh sieve. The cells were cultured in a growth medium consisting of DMEM-F12 (Gibco, Grand Island, NY, USA) supplemented with 20% FBS and 10% heat-inactivated horse serum (Gibco, Grand Island, NY, USA). Two hours later, the cell supernatant was transferred to a new flask and the cells began to adhere after 48 h. Myoblasts within four generations were used for subsequent studies. Differentiation of the myoblasts was carried out in a medium containing 2% horse serum in DMEM-F12. The differentiation was observed at 0, 72, and 120 h after differentiation.

### 4.3. Gene Expression Analysis

The total RNA of cells and tissue samples was extracted using a Trizol reagent (Takara, Dalian, China) according to the manufacturer’s instructions. The extracted RNA pellets were resuspended in DEPC treated deionized water. RNA concentration and quality were measured via NanoDrop 2000 spectrophotometry (Thermo Scientific, Waltham, MA, USA), and an optical density value of 260/280 for the samples between 1.8 and 2.0 was used for further experiments. Reverse transcription reagent kits (Takara, Dalian, China) were used to remove genomic DNA (gDNA Eraser, up to 1 mg/reaction, 2 min, 42 °C and to reverse transcribe (Master Mix, 37 °C for 15 min, 85 °C for 5 s) the RNA samples. Quantitative real-time PCR (qRT-PCR) assessment was performed using the Step One Plus Real Time PCR System, and fluorescence was detected using SYBR Green (Roche, Mannheim, Germany) in a reaction volume of 20 μL. The sequences and GenBank accession numbers of the primers used for gene amplification are listed in Table S1. The relative quantification of the target gene expression levels was normalized to glyceraldehyde-3-phosphate dehydrogenase (*GAPDH*) using the 2^−∆∆*C*t^ method.

### 4.4. Cloning of p32

To investigate whether *p32* expresses in sheep muscle, and further obtain the coding sequence of *p32* in sheep, one pair of specific primers for the *p32*-CDS were designed using the Primer 5.0 software (Table S1). The sequence of *p32* was amplified using the muscle cDNA of Hu Sheep via PCR. The PCR conditions were as follows; 94 °C for 5 min, 35 cycles of 98 °C for 10 s, 60 °C for 45 s, 72 °C for 45 s, and 72 °C for 7 min. All PCR products were separated using 1.5% agarose gel. After purification, the target PCR products were cloned into a pClone007 Blunt Vector (TSINGKE Biological Technology, Beijing, China) and then transformed into *Escherichia coli* DH5a cells. The positive clones were randomly selected and sequenced at TSINGKE Biological Technology.

### 4.5. Small Interfering RNAs

The siRNAs targeting *p32* and the non-targeting control siRNA (NC siRNA) (Table S2) were purchased from Shanghai GenePharma (Shanghai, China). The sequences of the three siRNAs are listed in Table S2. Afterwards, the siRNAs were transfected into the sheep myoblasts using the Lipofectamine 3000 reagent (Invitrogen Life Technologies, Carlsbad, CA, USA), according to the manufacturer’s protocol. Firstly, the cells (2 × 10^5^) were seeded onto 6-well plates and incubated overnight. Then, 50 nM of *p32* siRNAs and NC siRNA were transfected into each well of the cells. Cells were harvested for qPCR 24 h after transfection and underwent Western Blot 48 h after being transfected.

### 4.6. Immunofluorescence

The expression of *p32* in myoblasts was examined by immunofluorescence analysis. Cells were seeded in a glass-bottom dish, fixed with ice-cold methanol for 20 min at room temperature, washed with PBS, and permeabilized for 10 min using 0.25% Triton X-100 (Sigma-Aldrich, St Louis, Missouri). Next, cells were blocked with an Immunol Staining Blocking Buffer (Beyotime, Shanghai, China) for 60 min at room temperature on a rocking platform and then washed with PBS. The primary rabbit anti-p32 antibody (1:100 dilution, Proteintech, Chicago, IL, USA) was added to the cells and incubated overnight at 4 °C. Afterward, the cells were washed three times with PBS, after which the secondary antibody, the 594-conjugated donkey anti-rabbit antibody (1:200 dilution, Abcam, Boston, MA, USA), was added to the cells and incubated for 2 h at room temperature in the dark. Finally, the nuclei of the cells were stained with 4′,6-diamidino-2-phenylindole (Beyotime, Shanghai, China) for 10 min, and cell fluorescence was examined using a confocal laser scanning microscope (Zeiss LSM 710 META, Mannheim, Germany).

### 4.7. Western Blot Analysis

The total protein was prepared using a protein lysis buffer (Radio Immunoprecipitation Assay; Beyotime, Shanghai, China) supplemented with phenylmethanesulfonyl fluoride (PMSF; Beyotime, Shanghai, China).

Then, the proteins were denatured in a sodium dodecyl sulphate (SDS) gel-loading buffer for 10 min at 98 °C. After protein quantification, samples (20 mg/lane) were loaded on 12% SDS polyacrylamide gel electrophoresis (SDSPAGE) and electro-transferred to a polyvinylidene fluoride (PVDF) membrane (Millipore; Billerica, MA, USA). The membranes were blocked in a blocking buffer (5% BSA in Tris-buffered saline containing 0.1% Tween 20) for 2 h at room temperature, and then incubated at 4 °C overnight, with corresponding primary antibodies to *ATCB* (1:2000, Bioss, Beijing, China), *p32* (1:1000, Proteintech, Chicago, IL, USA), *AMPK* (1:500, Bio-Rad, Hercules, CA, USA), *p-AMPK* (Thr172) (1:1000, Affinity, Boston, MA, USA), *LKB1* (1:2000, Bioss, Beijing, China), *PCNA* (1:1000, Affinity, Boston, MA, USA), *BAX* (1:1000, CST, Boston, MA, USA), *Bcl-2* (1:1000, CST, Boston, MA, USA), *p-mTOR* (Ser2448) (1:1000, CST, Boston, MA, USA), *mTOR* (1:1000, CST, Boston, MA, USA), Raptor (1:2000, Affinity, Boston, MA, USA), and *p-Raptor* (Ser792) (1:2000, Affinity, Boston, MA, USA), *MyHC* (1:1000, Proteintech, Chicago, IL, USA), *GCS1* (1:1000, Proteintech, Chicago, IL, USA). After washing with TBST, membranes were incubated with the peroxidase-conjugated secondary antibody (horseradish peroxidase (HRP)-labeled Goat Antirabbit IgG or HRP-labeled Goat Anti-Mouse IgG) for 60 min at room temperature. The membranes were visualized using an enhanced chemiluminescence detection system (Fijifilm, Tokyo, Japan), and the chemiluminescence intensity of each protein band was quantified using Image J software (National Institutes of Health, Bethesda, MD, USA).

### 4.8. Flow Cytometry Analysis

Apoptosis was detected by the Annexin V-FITC/PI double staining method [25]. Myoblasts were washed twice with DPBS and resuspended in 100 μL of one labeling buffer containing PI and FITC conjugated Annexin V. After incubation for 15 min in the dark at room temperature, the stained cells were sorted using a flow cytometer (BD Biosciences, Franklin Lake, NJ, USA).

The cell cycle was also detected by flow cytometry analysis. Collected cells were fixed in 70% ice-cold ethanol overnight at −20 °C. After a wash in DPBS, the cells were incubated with 0.5 mg/mL RNase for 30 min at 35 °C and stained with 0.025 mg/mL PI for 10 min. Finally, the cells were evaluated by flow cytometry analysis for identifying cells at different stages of the cell cycle. Data were collected from at least 10,000 cells for each sample.

### 4.9. ELISA Assay

Cells and the culture medium collected to determine glucose concentration, lactate concentration, and cellular ATP level were quantified with an ELISA assay using commercial ELISA kits, according to the manufacturer’s instructions (Kmaels Co., Ltd., Shanghai, China).

The cells were trypsinized and collected into a tube. The cells were collected by centrifugation at 600 g for 5 min at 4 °C, and the supernatant was carefully aspirated, while ensuring that as few cells as possible were aspirated. Then, the cells were washed once with PBS. After absorbing the supernatant, add the lysate according to the ratio of adding 100 μL of lysate per 2 million cells (if the lysis is insufficient, increase the amount of lysate to 150 or 200 μL) and resuspend the pellet and add ice. The bath was then lysed for 15 min. The cellular ATP levels were analyzed by ELISA, following the instructions for ATP (DRE-S077, Kmaels Biotech, Shanghai, China). The culture medium was centrifuged at 3000 g for 10 min, after which the supernatant was collected and stored at –20 °C. Glucose concentration and lactate concentration were analyzed by ELISA, following the instructions for the Sheep Glucose (DRE-S1205, Kmaels Biotech, Shanghai, China) and Lactate (DRE-S1219, Kmaels Biotech, Shanghai, China) ELISA kits by Kmaels Biotech Co., Ltd. (Shanghai. China). First, the wells on a detection plate were designated as standard wells, sample wells, and blank wells. Next, 50 µL of different concentrations of the standard were added into the standard wells, and the 10 µL of blood samples and 40 µL of sample dilutions were added into the sample wells. Second, 50 µL of the horseradish peroxidase (HRP) labeled detection antibodies were added into each well and incubated at 37 °C for 60 min. Next, after washing the wells five times using the washing solution, 50 µL of substrates A and B were added into each well and incubated in the dark at 37 °C for 15 min. Finally, 50 µL of the stop solution was added, and the OD values were detected at a wavelength of 450 nm for 15 min. The coefficients for the variation of the inter- and intra-assay CV were less than 15% during the detection process.

The cells were trypsinized and collected into a tube. The cells were collected by centrifugation at 600 g for 5 min at 4 °C, and the supernatant was carefully aspirated, while ensuring that as few cells as possible were aspirated and washed once with PBS. After absorbing the supernatant, the lysate was added according to the ratio of adding 100 μL of lysate per 2 million cells (if the lysis is insufficient, the amount of lysate was increased to 150 or 200 μL), the pellet was resuspended, and ice was added. The bath was then lysed for 15 min.

### 4.10. EDU Assay

The EDU assay kit was purchased from KeyGEN BiolTech (Jiangsu, China), and all procedures were done in accordance with the manufacturer’s instructions.

### 4.11. Isolation of Mitochondria

The Cell Mitochondria Isolation Kit was purchased from Beyotime (Shanghai, China), all procedures were done in accordance with the manufacturer’s instructions.

The cells were washed once with PBS and digested with Trypsin-EDTA Solution (Beyotime, Shanghai, China), 100–200 g. Then, the cells were collected by centrifugation at room temperature for 5–10 min. The cell pellet was then gently resuspended in cold PBS, and a small number of cells were taken for counting, and the remaining cells were 600 g, and the cells were pelleted by centrifugation at 4 °C for 5 min and the supernatant was discarded. The precipitation was added and 1–2.5 mL of mitochondrial separation reagent added with PMSF to 20–50 million cells to suspended the cells. After suspension, the cells were placed in ice bath for 10–15 min. Then, the cell suspension was transferred to a suitable size glass homogenizer and homogenized for about 10–30 times. The cell homogenate was centrifuged at 600 g for 10 min at 4 °C. The supernatant was transferred to another centrifuge tube and centrifuged at 11,000× *g* for 10 min at 4 °C The supernatant was cytoplasmic protein without mitochondria, and the precipitation was mitochondria. The supernatant and the precipitation were used for Western blot analysis.

### 4.12. Statistical Analysis

All data were analyzed using SPSS software (version 20.0) by an independent Student’s *t*-test or one-way analysis of variance (ANOVA) with Tuckey post hoc analysis. For all analyses, *p* < 0.05 was considered statistically significant. All experiments were carried out in triplicate. All values were expressed as the mean ± SEM.

## 5. Conclusions

In summary, this study reveals that p32 is necessary for the muscular development of sheep. p32 is highly expressed in fetal sheep longissimus muscle and quadricep muscle tissues. The expression of p32 was increased with myoblast differentiation in vitro. The interference of p32 in sheep myoblast shifted the energy metabolism from OXPHOS towards glycolysis in vitro, which, in turn, decreased ATP. This led to an increase in the expression of LKB1, which activated the AMPK pathway, resulting in inhibited myoblast differentiation, proliferation, and increased apoptosis.

## Supplementary Materials

Supplementary materials can be found at https://www.mdpi.com/1422-0067/20/20/5161/s1. Figure S1. The expression of the p32 in mitochondria and in cytoplasmic protein. Figure S2. The total fusion index of myoblasts after interference p32. Table S1. Details of primer sequences used for this study. Table S2. Details of siRNAs squences used for this study.

## Abbreviations

MDPI	Multidisciplinary Digital Publishing Institute
DOAJ	Directory of open access journals
TLA	Three letter acronym
LD	Linear dichroism
C1QBP	Complement 1q binding protein C
OXPHOS	Oxidative Phosphorylation
AMPK	Activated AMP-activated protein kinase
MEF	Mouse embryo fibroblast
ATP	Adenosine triphosphate
LKB1	Liver Kinase B1
mTROC1	Rapamycin-sensitive mTOR complex 1
CDS	Sequence coding for amino acids in protein
CASP3	Caspase-3
CASP9	Caspase-9
GCS1	Endoplasmic reticulum anchored enzyme mannosyl-oligosaccharide glucosidase I
EDU	5 – ethynyl – 2′ - deoxyuridine
DAPI	4′,6-diamidino-2-phenylindole
DEPC	Diethyl pyrocarbonate
si-NC	Non-targeting control siRNA
PBS	Phosphate buffer saline
MyHC	Myosin heavy chain
MyoD1	Myogenic differentiation 1
MyoG	Myogenin
ACTB	β-actin
MyH7	Myosin heavy chain 7
VDAC1	Voltage dependent anion channel 1
