# Supplementary material for: The Expression Pattern of p32 in Sheep Muscle and Its Role in Differentiation, Cell Proliferation, and Apoptosis of Myoblasts
Source: Int J Mol Sci. 2019 Oct 18;20(20):5161. doi: 10.3390/ijms20205161 (PMC6829534; doi:10.3390/ijms20205161)
Supplement: Supplementary file 1 [file ijms-20-05161-s001.pdf]

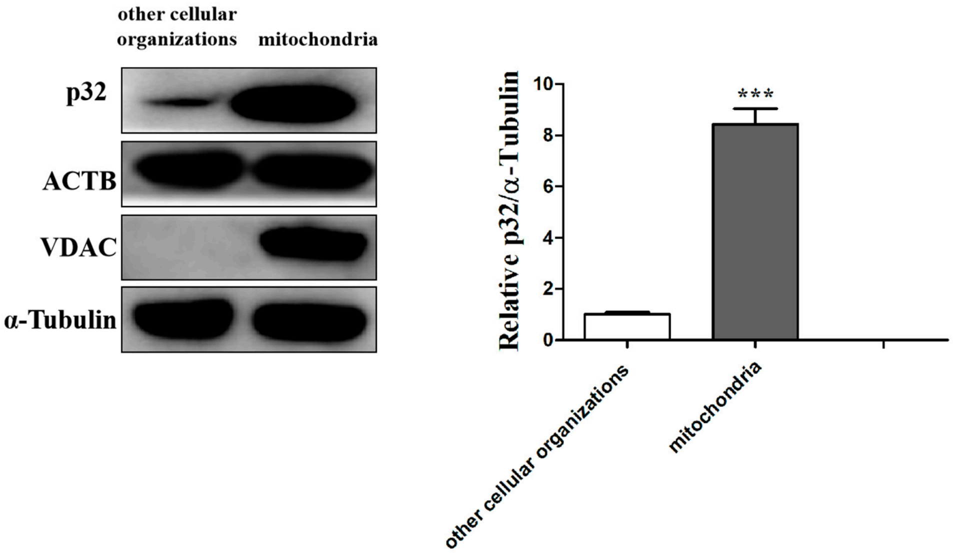

Figure S1. The expression of the p32 in mitochondria and in cytoplasmic protein without mitochondria was detected by Western blot. The data are represented as the mean ± S.E.M (n = 3). *** denote the significant and extremely significant difference between the two groups at $p < 0.001$.

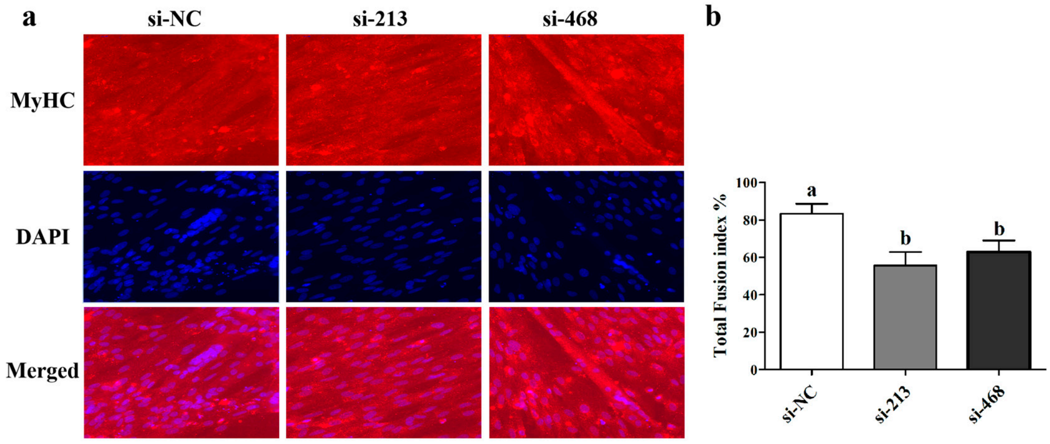

Figure S2. The total fusion index of myoblasts after interference p32 was detected using Immunofluorescence of MyHC. Sheep myoblast and myotube stain with the MyHC antibody (red) and nuclear stain DAPI (blue). Scale bar = 50 μm. Results are expressed relative to the si-NC as mean values ± SEM (n = 3). a, b, c: different letters denote statistically significant differences within each group; $p < 0.05$.

Table S1. Details of primer sequences used for this study.

Target Gene	Primer Sequence (5′-3′)	Product Size (bp)	Accession Number
**GAPDH**	F: GTCAAGGCAGAGAACGGGAA<br>R: GGTTCACGCCCATCACAAAC	232 bp	NM_001190390.1
**LKB1 (STK11)**	F: GTGAAGGAGGTGCTGGACTC<br>R: TCCTTCTTCACGTTGGCCTC	107 bp	XM_004008774.3
**AMPK (PRKAA1)**	F: TTGCGTGTTCGGAGGAAGAA<br>R: GGCGTAGCAGTCCCTGATTT	146 bp	XM_012156760.1
**BAX**	F: GTGTCTGAAGCGCATTGGAG<br>R: TCGGAAAACATTTCAGCCGC	120 bp	XM_004015363.3
**Bcl-2**	F: CGCATCGTGGCCTTCTTT<br>R: CGGTTCAGGTACTCGGTCATC-	113 bp	DQ152929.1
**CASP3**	F: TCAGGGAAACCTTCACGAGC<br>R: CCTCGGCAGGCCTGAATAAT	274 bp	XM_015104560.1
**CASP9**	F: GCCAAGCCAAGGAAAACTCG<br>R: CACGGCAGAAGTTCACGTTG	236 bp	XM_012187488.2
**PCNA**	F: AGTGGCGTGAACCTACAGAG<br>R: GCCAAGGTGTCCGCATTATC	203 bp	XM_004014340.3
**P53**	F: ATGGAAGAATCGCAGGCAGAACTC<br>R: AATACGTGCAGGTCACAGACTTGG	343 bp	X81705
**p32**	F: GCTGTAGCGGACTGCACACC<br>R: CAATTCAGGCTCCTGCTCCTCAG	281 bp	XM_004012589
**p32-cDNA**	F: GCCAAAATGTTCCAGCTGCT<br>R:<br>CTGGCTTTTGACAAAACCTTTGAGGT<br>CTTC	856 bp	XM_004012589
**MYOG**	F: GCAGCGCCATCCAGTACATA<br>R: GACTGCAGGAGGCACTATGG	131 bp	NM_001174109
**MyH7**	F: GTTTGAAAAGCCAAGCCGC<br>R: TGAGGTCAAAAGGCCTGGTC	120 bp	XM_012129251
**MyHC**	F: CTTCGTGGCGGACCCTAAG<br>R: CAGTTACTGTCGCCCCAGCT	101 bp	AB058898
**MyOD**	F: CCCTGGTGACTTCAGCTGTT<br>R: TACAAAGTCCCTGTCGCACC	151 bp	AF184163

Table S2. Details of siRNAs sequences used for this study.

Name	Sequence (5′-3′)
**Si-213**	Sense: GCACACCGAAGGAGACAAATT<br>Anti-Sense: UUUGUCUCCUUCGGUGUGCTT
**Si-468**	Sense: GCAGGAGCCUGAAUUGACATT<br>Anti-Sense: UGUCAAUUCAGGCUCCUGCTT
**Si-689**	Sense: GCUUAUACGACCACCUAAUTT<br>Anti-Sense: GCUUAUACGACCACCUAAUTT
**Si-NC**	Sense: UUCUCCGAACGUGUCACGUTT<br>Anti-Sense: ACGUGACACGUUCGGAGAATT
